# What Makes You Can also Break You, Part III: Mitochondrial Permeability Transition Pore Formation by an Uncoupling Channel within the C-Subunit Ring of the F1FO ATP Synthase?

**DOI:** 10.3389/fonc.2014.00235

**Published:** 2014-09-03

**Authors:** Christos Chinopoulos, Gyorgy Szabadkai

**Affiliations:** ^1^Department of Medical Biochemistry, Semmelweis University, Budapest, Hungary; ^2^Department of Cell and Developmental Biology, Consortium for Mitochondrial Research, University College London, London, UK; ^3^Department of Biomedical Sciences, University of Padua, Padua, Italy

**Keywords:** commentary, mitochondrial transition pore, F1Fo-ATPase, PTP, uncoupling channel

In the hunt for identifying the structural elements of the mitochondrial transition pore, the recent paper by Alavian et al. proposes that the pore is formed by an uncoupling channel within the c-subunit ring of the F_1_F_O_ ATP-synthase complex (1), reaffirming previous results (2, 3) and providing exciting additional details about the potential molecular mechanism controlling pore formation.

The paper compellingly demonstrates that purified reconstituted c-subunit rings in liposomes exhibit channel activity with multiple subconductance states that are sensitive to inhibition by adenine nucleotides, recombinant F_1_ beta-subunit protein, and anti-c-subunit antibodies. Channel activity matched that of the mitochondrial megachannel, now known as being the permeability transition pore (PTP) (4). Moreover, by fluorescently labeling c-subunit rings in living cells, they were able to monitor the opening and closing of the ring in response to Ca^2+^ and the PTP inhibitor, cyclosporin A. By introducing site-specific mutagenesis of highly conserved glycines within the N-terminus of the α-helical region of the c subunit, which are responsible for the tight packing of the ring, thus making it looser, they achieved an increase in channel conductance by an order of magnitude, accompanied by loss of sensitivity to ATP. Finally, they propose that the physical uncoupling of F_1_ from F_0_ triggers the increase in c-subunit pore conductance, providing a mechanism for induction of the permeability transition.

The papers by Alavian et al. (1) and Bonora et al. (2) provide strong evidence that the c-subunit ring comprises the long-sought pore of the permeability transition and certainly opens the way for further genetic, structural, and eventually pharmacological testing of its role and mechanism in physiological and pathological processes. At the same time, one can suspect that the debate on some previously described properties of pore formation in mitochondria is not entirely over.

Indeed, it is not the first time that purified mammalian c subunits have been reconstituted in lipid bilayers and voltage-clamped; and what might appear troubling is that there are stark differences in the electrophysiological signature of the c-subunit conductances shown in Ref. (1) compared to what have been shown in earlier works by McGeoch et al. (5–7).

First, in the work of McGeoch et al., AMP *activated* the subunit-c conductance, while ATP (and other nucleotides) had no effect on the current, as opposed to the results by Alavian et al., where the purified c-subunit channel activity was equally *attenuated* by ATP, ADP, or AMP. Alavian et al. inferred that some ATP sensitivity is localized directly within the c-subunit itself; however, the c-subunit ring of the F_1_F_O_ ATP-synthase complex has not been reported to have access to ATP; accordingly, by using a bioinformatic approach, the c-subunit protein is not predicted to exhibit binding residues for any nucleotides (8).

Second, McGeoch et al. reported that the c-subunit current was extremely sensitive to *inhibition* by Ca^2+^ (Figure 1A), demonstrating the closure of subunit c pores due to the cooperative effect of at least four Ca^2+^ ions per ring (7). In contrast, Alavian et al. showed that the current mediated by reconstituted c subunits in lipid bilayers is *not* sensitive to Ca^2+^, and it is *induced* by Ca^2+^ when measured in submitochondrial vesicles (1), as expected by the PTP (9). McGeoch proposed that the Ca^2+^ ions mediate the closure of the ring by binding on sites in the lumen of the channel, which forms a pore with a hauntingly similar size to what has been proposed for the PTP (~2–3 nm) (Figure 1C). Indeed, when presented with a water surface, c rings change their hydrogen bonding from an α-helix to β-sheet-like configuration and move away from previous associations with lipids to interact with water surface molecules (10).

**Figure 1:**
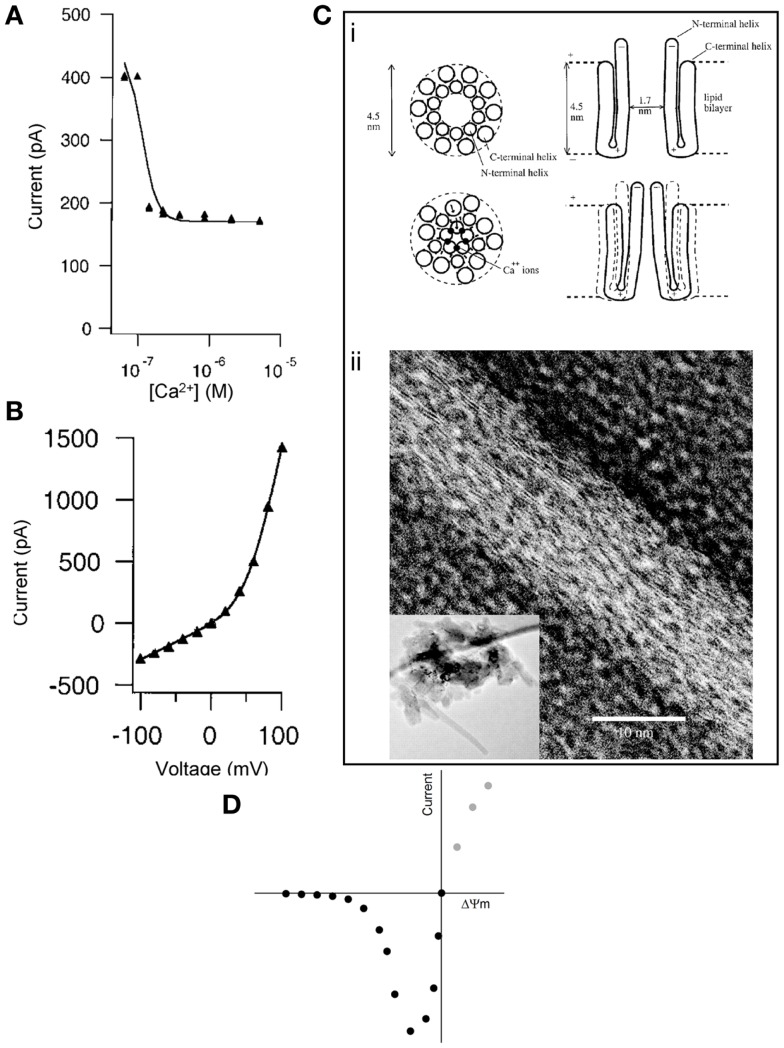
**(A)** Inhibition of subunit c pore Na^+^ current by Ca^2+^ (added to bath) reconstituted in liposomes. Conditions: 60 mV, ionic strength = 0.06 M, [Na^+^] = 50 mM, [Mg^2+^] = 15 mM. **(B)** Current-voltage plot of subunit c pore current as constant ionic strength of 0.2 M. Both panels were reproduced from McGeoch et al. (7) by permission from Elsevier. **(C)** (i) Concept for the Ca^2+^ control of the mammalian subunit c pore conductance. Reproduced from McGeoch et al. (7) by permission from Elsevier. (ii) Transmission electron microscopy (inverted image) of a tube whose surface is covered by strands of subunit c with a lateral spacing of 3.7 Å amidst circular structures that could be assembled channels of subunit c of 3 nm diameter. The inset image shows a lower magnification of the entire pile of collapsed tubes on the grid and in their center are crystals. Reproduced from McGeoch and McGeoch (10) (free via Creative Commons). **(D)** Hypothetical current-voltage plot of the PTP during patch-clamp in symmetrical solution. Intersect is at 0 mV, 0 pA.

It is likely that these discrepancies are due to the use of purified protein complexes using different sources and protocols, exposing diverse binding sites for these regulators. Indeed, as the authors pointed out, the high-affinity site(s) of the PTP for adenine nucleotides as well as the Ca^2+^ binding sites are likely to be located outside the c ring (1), and in the case of Ca^2+^, this binding may well overcome the effect of Ca^2+^ inside the c subunits. Furthermore, based on the available data, an important role for interactions between more than one c ring in the formation or regulation of the PTP cannot be formally excluded, which might even be compatible with the results of Giorgio et al. proposing dimer formation by whole F_1_F_O_ ATP-synthase complexes as a crucial event (11), reviewed in Ref. (3). In our view, while it might seem difficult to achieve with current methodologies, only the reconstitution of recombinant complexes, or solving the structure of the open and closed pore at sufficiently high resolution would clarify these properties of a pore formed by c subunits.

The same methodological limitations might result in a third controversial issue. In the work of McGeoch et al., the current-voltage plot of the c-subunit pore exhibited *outward* rectification (Figure 1B), as opposed to an *inward* rectification claimed by Alavian et al. [although the Figure 1B in Ref. (1) does not suggest any rectification]. Regarding the “voltage-sensing” of the c-subunit current in proteoliposomes (1), we must first distinguish this from the “voltage dependence” of the PTP in mitochondria (12). The probability of PTP opening is inversely related to the magnitude of the proton electrochemical gradient across the inner mitochondrial membrane. The module conferring sensitivity of the pore to this gradient has never been identified. Likewise, the mitochondrial megachannel that has been electrophysiologically investigated in mitoplasts also exhibits voltage dependence, showing a higher probability of opening as the potential across the inner mitochondrial membrane (ΔΨm) becomes less negative (13). If the macroscopic current of the reconstituted c ring in liposomes were to exhibit the same kind of voltage dependence as the PTP in mitochondria or the megachannel in mitoplasts, patch-clamping of the c ring in “symmetrical” solution (an experimental condition in which the solutions across the two sides of the patched membrane contain equal concentrations of the major permeant ions) should yield a current-voltage relationship similar to that depicted in Figure 1D. Briefly, the magnitude of the absolute current through the c ring would increase as the voltage became less negative; when the voltage would closely approach the “0” value (intersect), the current would also tend to become zero, simply due to the symmetrical solution. The magnitude of the current at positive potentials (gray circles) cannot be predicted because mitochondria cannot achieve a positive value of ΔΨm. However, the electrophysiological signatures of the reconstituted c ring shown in Ref. (1) and (7) and of the megachannel reviewed in Ref. (13) are far from the expected current-voltage plot shown in Figure 1D. Altogether, the discrepancy of findings regarding voltage dependence along with Ca^2+^ and nucleotide sensitivity argue that the reconstituted c ring in proteoliposomes, devoid of all regulatory and other modules, is bound *not to* exhibit the same properties as the PTP. Likewise, the primary current through the subunit c pore reported by McGeoch was cationic, with anionic current not being observed (5), while the mitochondrial megachannel exhibited low anion selectivity with rare switches to cation selectivity (13); Alavian et al. did not investigate anion selectivity. In our opinion, it is difficult to imagine cation vs. anion selectivity of a 2–3 nm diameter channel, which is known to be non-selective for solutes with a molecular weight of up to 1,500 Da (4) and maximum open probability near 0 mV (13). To the above divergence of findings, the conflicting effect of *N*,*N*′-dicyclohexylcarbodiimide (DCCD) can also be added; DCCD is known to interact with subunit c and inhibit the entire operation of the F_1_F_O_ ATP-synthase complex (14); accordingly, DCCD was found to completely inhibit the conductance of the c-subunit channel (7), but on the other hand, DCCD is an atypical *inducer* of the permeability transition (15).

In the same way, the apparent complexity of the molecular entities embedding the actual pore also forecast the difficulties to study the behavior of permeability transition in living cells and tissues. Here, again the authors excel to demonstrate pore opening using classical inducers such as Ca^2+^ ionophores and pro-oxidants, but in our experience, it is excruciatingly difficult to generate reproducible data in many cellular models using these tools. Thus, a further important advance of this study is paving the way for novel genetic approaches (e.g., expressing constitutively open mutant pores, or silencing subunits) for live cell/organism studies. In this respect, an important claim of the present model is the uncoupling of the F_1_ and F_0_ subunits as the main trigger of pore opening, which should be amenable to interventions increasing or decreasing the sensitivity of the pore to pathophysiological stimuli. As a last point, it is also important to realize that the fundamental regulatory modules of the PTP (such as the voltage sensor, Ca^2+^, and nucleotide-binding sites) are yet to be elucidated; they may turn out to be targets more amenable to pharmacological or genetic manipulations for combating diseases in which the PTP is known to have a role.

## Conflict of Interest Statement

The authors declare that the research was conducted in the absence of any commercial or financial relationships that could be construed as a potential conflict of interest.
